# Harnessing 3D microarchitecture of pterosaur bone using multi-scale X-ray CT for aerospace material design

**DOI:** 10.1038/s41598-025-88257-0

**Published:** 2025-02-17

**Authors:** Nathan Pili, Tristan J. Lowe, Lee Margetts, Kevin Pickup, William I. Sellers, Emma L. Nicholls, Philip J. Withers, Phillip L. Manning

**Affiliations:** 1 Department of Earth and Environmental Sciences, The University of Manchester, Manchester, M13 9PL UK; 2 Department of Materials, Henry Royce Institute, The University of Manchester, Manchester, M13 9PL UK; 3 Department of Mechanical and Aerospace Engineering, The University of Manchester, Manchester, M13 9PL UK; 4 BAE Systems, Samlesbury Aerodrome, Balderstone, BB2 7LF UK; 5 Oxford University Museum of Natural History, Parks Road, Oxford, OX1 3PW UK; 6 Natural History Museum Abu Dhabi, Jacques Chirac Street, Abu Dhabi, UAE

**Keywords:** Engineering, Palaeontology

## Abstract

Pterosaurs were the largest animals to have achieved powered flight in the history of life on Earth, possessing wingspans akin to some modern light aircraft. Vertebrate fossils have shown their potential to retain information on the chemical, physical, and mechanical properties of precursor bone. However, the fossil record is not a traditional source of inspiration for engineers to create palaeo-bioinspired designs. To explore its potential, this study has imaged the three-dimensional porosity of pterosaur bone intending to inspire and improve the mechanical properties of aerospace materials. Historically, two-dimensional histological analysis has resolved fine-scale structures in fossilised bone, which damages the sample. By applying advanced X-ray imaging techniques in this study (using Image Quality Indicators) we show it is possible to non-destructively resolve/verify the microarchitecture of pterosaur bone not previously seen in three dimensions. Pterosaur bone porosity has helped map the macroscopic stresses of this biomaterial but ultimately presents an opportunity to inspire advanced manufactured materials. This microarchitecture of bone offers a unique geometry where self-healing materials with internal monitoring systems can be developed. The iterative process of Darwinian natural selection has evolved multiple engineering solutions that can be reverse engineered to solve challenges facing industry in the 21st Century.

**Supplementary Information:**

The online version contains supplementary material available at 10.1038/s41598-025-88257-0.

## Introduction

Natural form has served as an inspiration for novel engineering solutions for centuries^1^. Leonardo Da Vinci’s drawings of a piloted aircraft drew bioinspiration from the anatomy of bird wings^1^. More recently, the term ‘biomimicry’ was coined by Otto Schmitt^2,3^, and nature-inspired design can be found in many engineered materials/solutions throughout the 20th and 21st Centuries. Velcro was inspired by plants^4^, self-cleaning car designs are rooted in giant lotus leaves^5^, while aircraft are made ever more aerodynamic and efficient through the study of bird wings^6^. However, the species present today have been estimated to represent less than 1% of the diversity of life throughout Earth’s history^7–9^. Extinct species were numerous and possessed their own suite of adaptations to each environmental niche they occupied, but changes to climate and environment helped drive extinction events that were an inevitable consequence of evolution, regularly resetting this iterative bioengineering clock^10,11^. It is possible that human engineering can utilise the fossil record to unlock the potential of evolution’s solutions to form and function^12^. This past fossil repository of ‘palaeobiomimicry’ may well contain unrealised potential for innovative solutions in the future.

Humans achieved powered flight in the last hundred years^13^, but natural selection has refined volant forms in various guises for hundreds of millions of years^14–17^. Pterosaurs were the largest animals known to have achieved powered flight during the evolution of life on Earth, reaching wingspans upwards of 10 m^18,19^, identifying them as prime candidates for palaeobiomimicry research. Despite the challenges that come with studying and acquiring fossilised bone, their maximum wingspan is triple that of the largest living birds^20^. Therefore, these flying archosaurs had to solve multiple engineering challenges to get their enormous wingspan airborne, not least supporting their long wing membrane predominantly from a single digit (IV)^18^.

Powered flight in vertebrates is governed by multiple biological factors that influence the dynamics of flight. These factors include the shape and morphology of bone but also muscle properties, cardio-respiratory physiology and many more that are often impossible to resolve in extinct species given they are not preserved in the fossil record. However, one resolvable factor is bone architecture, the core skeletal scaffold for vertebrates and the focus of this study. In bone the composition, form and function of this living material is the product of the iterative process of natural selection^21^. Bone possesses multiple biochemical, physical and structural characteristics that aid in the function of vertebrate bodies, not just as a scaffold but as a factory for cell production and a store for nutrients and essential minerals^22,23^. Macroscopically, bone can be divided into cancellous (also known as trabecular bone) and cortical bone^24,25^. Cancellous bone is typically found near the proximal and distal ends of long bones and dominates small bones, the cortical is typically found covering the outer layer but is highly concentrated along the shaft of a bone^25^. Cancellous bone is dominated by spoke-like trabeculae that likely form and change according to the force environment in which they grow^26,27^. Many key adaptations are observable at the macro-scale and have been extensively studied^28–30^. Time-lapse X-ray tomography has been used to follow the progress of deformation from loading to failure^31^. Recent work on Finite Element Analysis (FEA) using X-ray tomography images of extant cancellous bone^32,33^ highlights the impact of microscopic adaptations on strength and resistance to damage^34^. However, few have focused on the analysis of the porosity of pterosaur cortical bone.

At the microscopic scale, there are structures in bone such as the Haversian canals that aid in nutrient transfer, growth, and maintenance^35–38^. These canals are microscopic structures (20–100 μm in diameter), allowing blood vessels and nerves to fulfil their roles in nutrient supply/exchange^39^. Gustafsson et al.^40^ showed that Haversian canals and surrounding cement lines possessed the ability to deflect horizontal cracks, therefore reducing the damage caused by microfractures. However, this study made many assumptions about the size and geometry of bone microarchitecture. Haversian canals are essential to biological function, filled with tissue and blood, but they may also be an exaptation, having evolved for biological function and simultaneously developing mechanical effects. The focus of this study, however, was not to accurately model the porosity, but instead to understand the architecture of the porosity, aiming to exploit the morphology for aerospace applications. If replicated correctly, such structures could be filled with sensors and/or self-healing liquids to create an engineered environment to support greater complexity on aircraft, or if filled with air they might create lightweight components that preserve strength. Therefore, if bone porosity was faithfully resolved through high-resolution imaging and then replicated in engineered components, this would create an abundance of design approaches for optimising component functionality. Resolving bone porosity and developing the use of such bio-inspired components is the focus of this study.

Fossilised remains of terrestrial vertebrates are very rare—particularly as complete or even partially articulated skeletons^41–43^. Even fewer fossils retain high-fidelity information, a consequence of the alteration that can occur both pre- and post-burial. One of the most effective ways to interrogate well-preserved specimens is through the use of X-rays that can penetrate rock and recover any resolvable porosity^44^. X-ray Computed Tomography (X-ray CT) uses a series of X-ray projections (radiographs) taken from different viewpoints to reconstruct a virtual volume that visualises the variation in attenuation coefficient of the scanned subject in three dimensions^45^. Some specialised X-ray CT scanners can achieve sub-micron resolution, but higher resolutions are generally associated with smaller regions of interest. This means to optimise data acquisition it is necessary to employ multi-scale imaging techniques. By imaging an entire bone using a medium-resolution X-ray CT scanner, it is possible to locate potential areas of interest to scan with higher-resolution instruments.

This study uses X-ray CT scans of a fossil pterosaur bone to resolve microstructural and macrostructural information to provide palaeo-bioinspiration for engineering components for the aerospace industry. To guarantee the academic and industrial value of these structures in engineering components, we focus on the vascular canals through a simple mathematical model using FEA. This model permitted stress analysis to measure the impact of the presence or absence of vascular canals, with the anticipation that they provide weight-savings through a reduction in the overall density and strength-improvements by crack termination.

We suggest that the adaptations that helped make pterosaurs highly successful animals can potentially provide novel engineering solutions to the many challenges facing the aerospace industry in the 21st century^46^. We aim to demonstrate through preliminary mathematical analyses and modelling that structures found in pterosaur bones can be reverse engineered to create efficient and lightweight components for industry.

## Results

### Computed tomography data

**Fig. 1:**
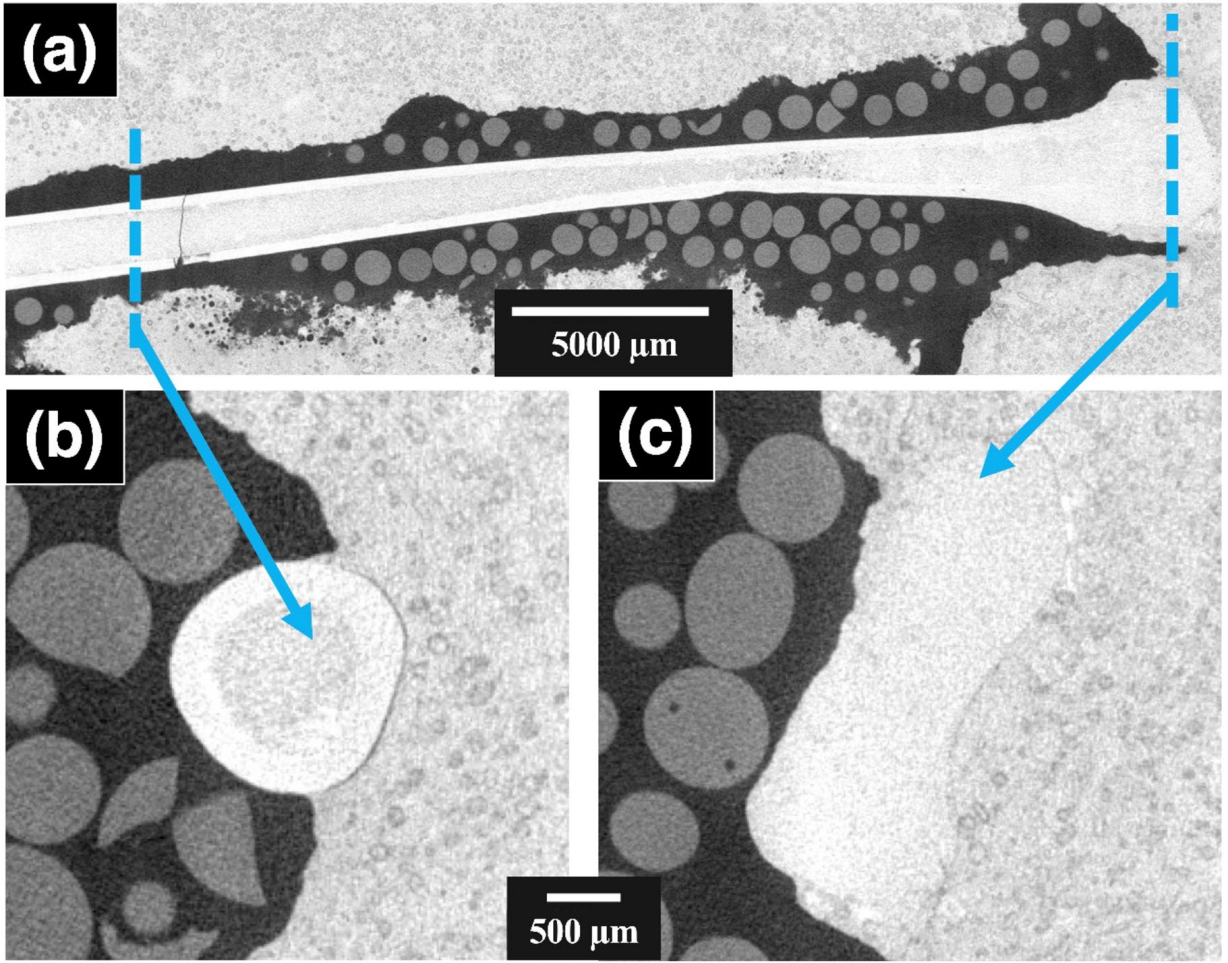
The raw reconstructed data for the *Rhamphorhynchus* sp. (OUMNH PAL-J.028331) wing bone scanned on the Nikon XT H. (**a**) is the whole bone along the longitudinal axis, (**b**) is an orthoslice near the distal end, which is exposed to air, and (**c**) is an orthoslice near the proximal end, which was predominantly encased in the sediment. The low-attenuating spheres are silicon balls that were used to reduce beam hardening effects, the central areas with high attenuation are bone structures, and the high-attenuating material beyond the bone is sedimentary matrix.

The macroscopic X-ray CT scan of a *Rhamphorhynchus* sp. (OUMNH PAL-J.028331) phalanx I of digit IV (Fig. 1a) shows the gross morphology of the bone. In areas where the fossil is exposed to air (Fig. 1b) it is possible to distinguish between the bone and the embedding sedimentary matrix because there is a high contrast between the fossil bone and matrix. However, the proximal head of OUMNH PAL-J.028331 is mostly encased in sediment (Fig. 1c) and the contrast subsequently decreases, likely as a function of the sediment attenuating X-ray photons.

The variation in X-ray beam attenuation resulted in more difficult visualisation, where the bottom two-thirds of the bone was easier to segment from the sediment, whereas the top third of the bone blended in with the sediment due to the additional noise and a lack of contrast. The final visualisation of the whole bone revealed a significant crack in the fossil.

**Fig. 2:**
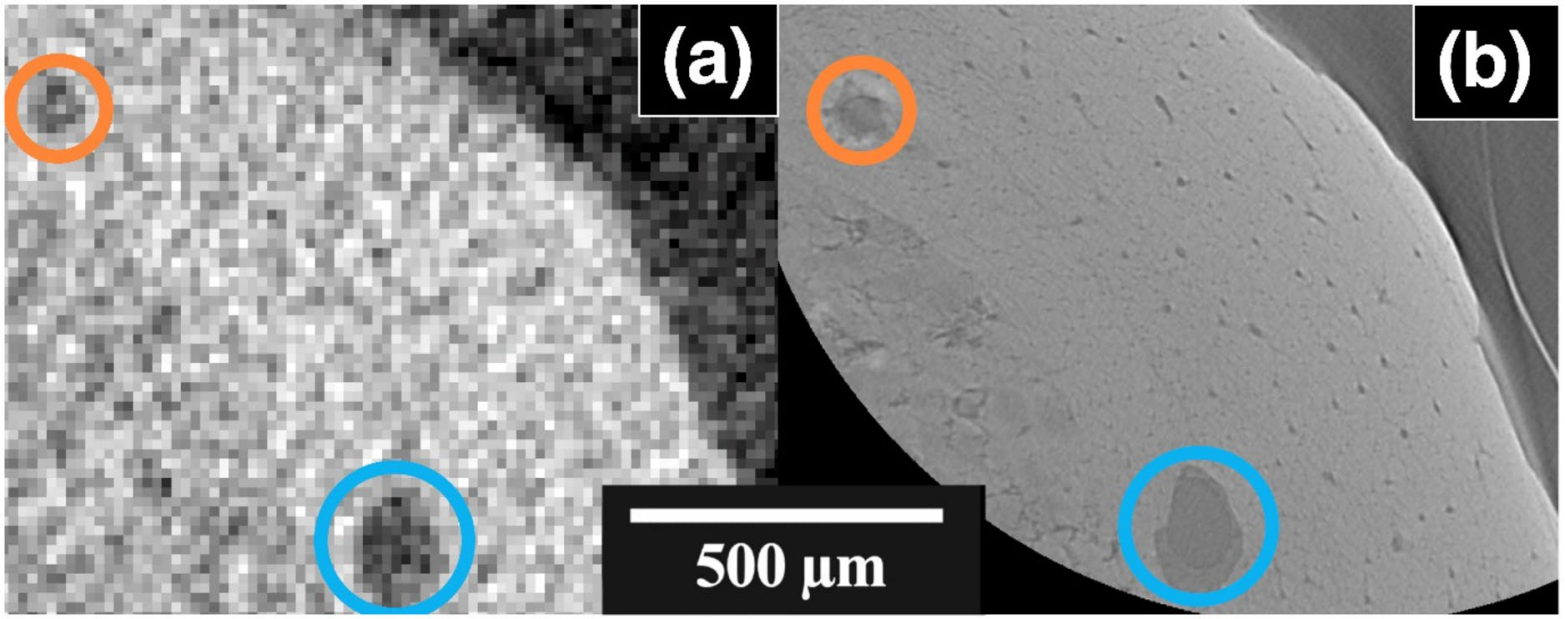
Orthoslices from scans of the same approximate area of cortical bone of a *Rhamphorhynchus* sp. (OUMNH PAL-J.028331) wing bone acquired using two different X-ray CT systems. (**a**) CT slice from the Nikon XT H (pixel size of 15 μm). (**b**) broadly the same region imaged using the Zeiss 520 Versa (pixel size 2.0 μm). Given the drastically different pixel sizes, it would be challenging to show exactly the same CT slice. The orange and blue circles highlight the same features in (**a**,** b**). Of note is the apparent homogeneity of the cortical bone in (**a**), whereas (**b**) shows canals throughout the cortical tissue. The thin line in (**b**) that sits in the air beyond the edge of the bone is the cellophane film used to protect the fossil during scanning (preventing glass beads from electrostatically clinging to the bone), the same film was not resolved using the Nikon XT H (**a**).

The region of interest scans of the OUMNH PAL-J.028331 were conducted on both the Nikon XT H (Figs. 1 and 2a) and the Zeiss 520 Versa (Fig. 2b) X-ray CT systems. After optimising the parameters to achieve enough flux through the sample, the smallest effective pixel size the Nikon XT H achieved was 15 μm (Fig. 2a). This resulted in a scan that covered a length of 27 mm of OUMNH PAL-J.028331 but generated a lot of noise in the final images. The cortical bone of OUMNH PAL-J.028331 was distinguishable from the sediment but was distorted by the presence of noise, which made it both difficult to segment and impossible to see smaller features, apart from large pores (though these could still be mistaken for noise if it were not for the higher resolution scans from the Zeiss 520 Versa instrument).

The Zeiss 520 Versa data covers a quarter of the cross-sectional area for a height of 1.9 mm at the distal end of OUMNH PAL-J.028331 (Fig. 2b). The X-ray CT system was again optimised for flux, giving a pixel size of 2 μm, largely limited by the length of exposure time and lack of transmission. This smaller pixel size shows greater detail in the large pores that were identified in the Nikon XT H data, but also shows additional features, such as the canals that were shown to make up ~ 16% of OUMNH PAL-J.028331 bone tissue volume. The average vascular diameter of each canal is 10.6 ± 2.0 μm, and there was an average frequency of 113 ± 14 mm^−2^ in the longitudinal direction. In this network of canals, 63% of nodes (connections or endpoints) were branching nodes and 37% were terminal nodes. This means that two-thirds of canals branched instead of ending.

**Fig. 3:**
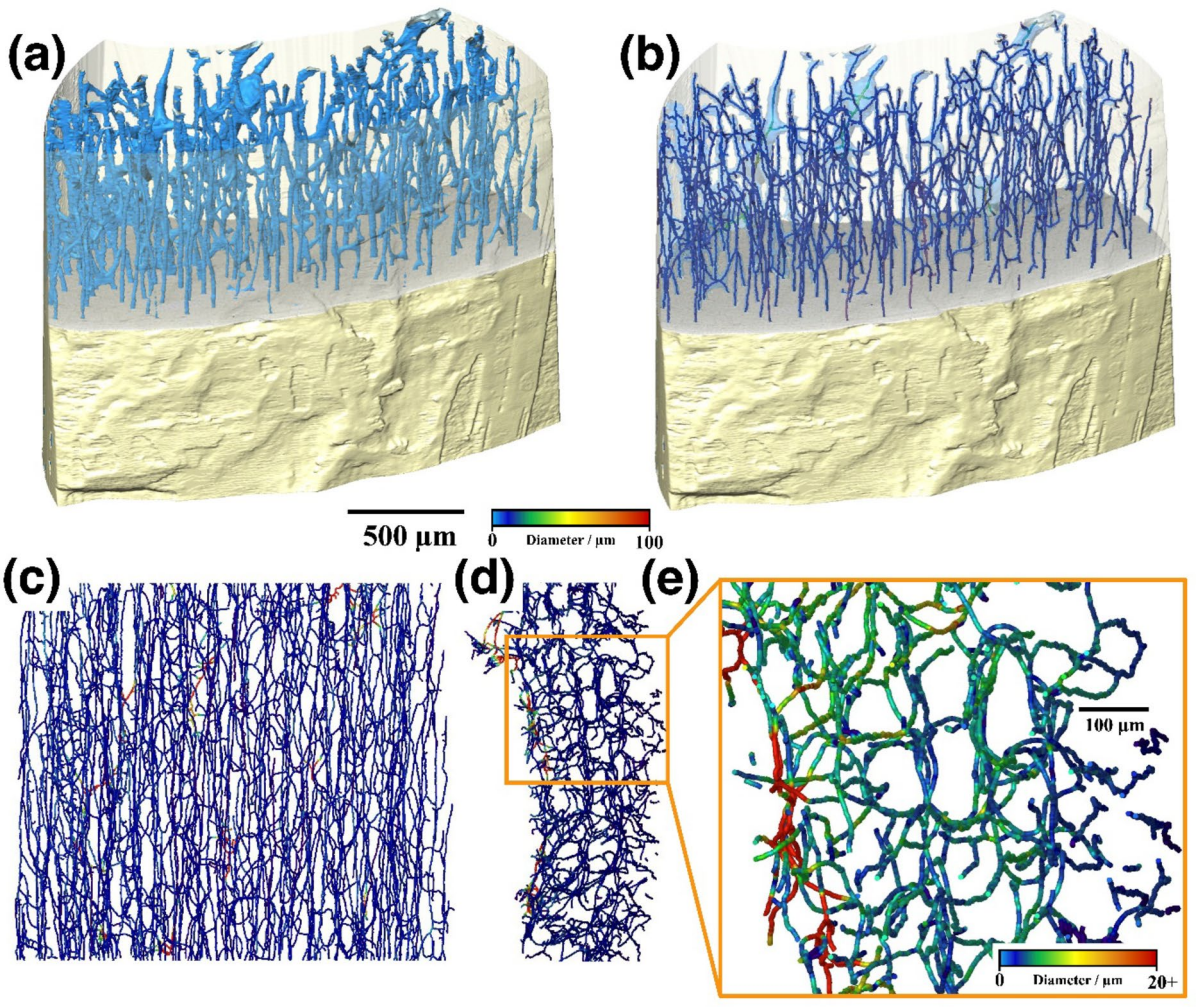
*Rhamphorhynchus* sp. (OUMNH PAL-J.028331) rendering (**a**) and canal skeletonised network visualisation (**b–d**) after segmentation from cortical bone. (**a**) Shows a 3D rendering of the CT scans, with the top half semi-transparent, showing the canals’ (blue) distribution throughout the bone. (**b**) Is similar to (**a**), but with the canal volume semi-transparent and instead shows the skeleton’s place in the canals. (**c**) Shows the canal network parallel to the bone’s longitudinal axis, and (**d**) shows the network perpendicular to the longitudinal axis where the bone’s outer edge is to the right. While most of the structure is homogeneous, there are a few areas where the canals get very large (the red areas in (**c**,** d**)). These are the large pores that can be seen in the CT data, which appear to connect to the rest of the canals. (**e**) Shows a magnified section of (**d**) with a smaller colour bar. There appear to be 3 different areas of the canal network. The inner section is filled with large pores (like those seen in Fig. 2), and the middle, appears to form a cellular pattern. The outer edge appears to have fewer canals, though this could be a consequence of some beam hardening.

The canals have a predominantly longitudinally trending orientation (Fig. 3a–c), although some are oriented radially and/or laminarly (Fig. 3d). The distribution and occurrence of canals appear to be uniform, but this drops off near the outer and inner surfaces of OUMNH PAL-J.028331 (Fig. 3d, e). There is little evidence in the orthoslices to suggest that the canals directly connect with the bone’s outer surface, although this could be a contrast issue.

### Finite element analysis

**Fig. 4:**
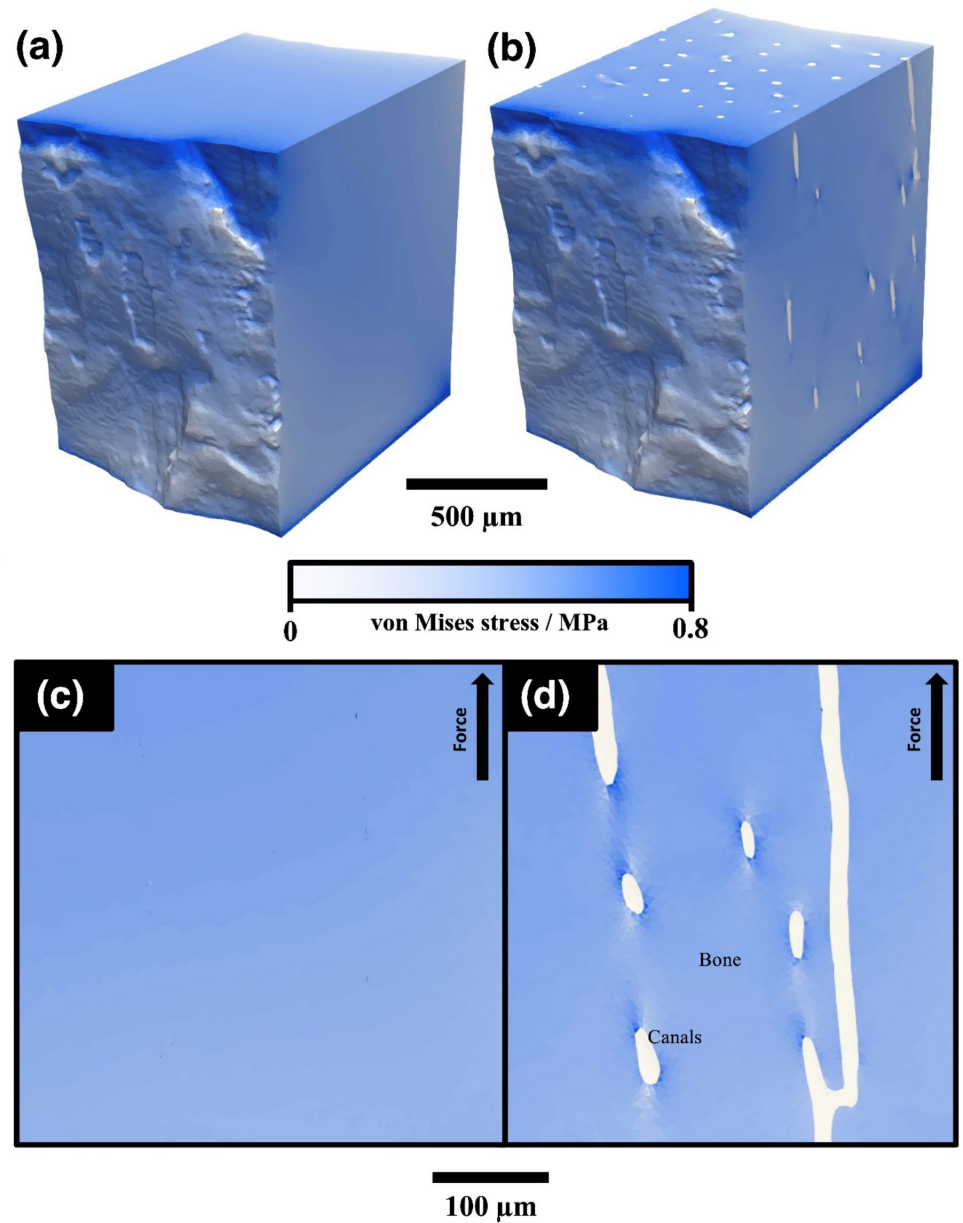
The distribution of von Mises stress for an axial (vertical) tensile strain of 2% predicted by the cortical bone FEA model constructed from the Zeiss 520 Versa scan of *Rhamphorhynchus* sp. (OUMNH PAL-J.028331) using MOOSE 3.0 ^47^. Canals here were modelled as (**a**,**c**) bone (i.e. no pores) and (**b**,**d**) liquid filled. The stress of the canals in this example is near zero, much like many areas of the cortical bone’s outer surface. (**c**,** d**) Show a magnified region from an XZ orthoslice, showing minor concentrations of stress local to the canals.

**Fig. 5:**
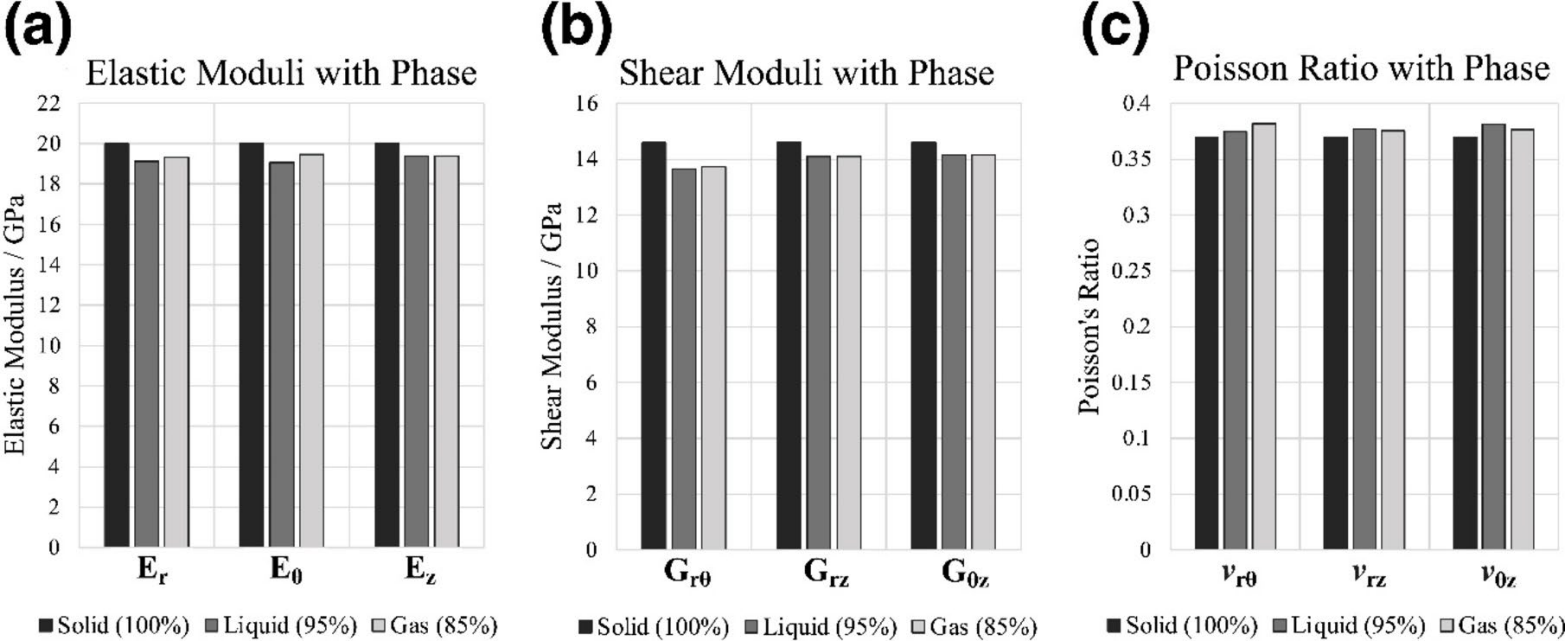
The effects of the canal phase (when filled by bone, liquid or air) on the various elastic constants in OUMNH PAL-J.028331. The final variables are given in cylindrical coordinates representing the homogenised properties of the cortical bone modelled as a cylinder. E_i_ is Young’s modulus in the i direction, G_ij_ is the Shear Modulus in the ij plane, and ν_ij_ is Poisson’s ratio in the ij plane (see Supplementary Table S1).

The finite element mesh constructed from the imaged porosity within OUMNH PAL-J.028331 allowed a view of the stresses affecting the longitudinally loaded bone around the canals using the FEA software MOOSE 3.0 (Fig. 4)^47^. The constant axial strain applied did not change the stress on elements far from the canals (the entire surface of the bone was largely unaffected by canal presence in Fig. 4). Figure 4 shows the distributions of the von Mises stresses, a scalar quantity which combines the 3 principal stresses at a point to give a value that indicates the propensity for the material to fractiure or deform at that location. The principal stresses, a vector quantity, were also measured and used to calculate the effective Young’s Modulus found in Fig. 5. The canals created areas of localised stress concentrations but did not substantially change the maximum von Mises and principal stresses experienced in the model.

If the phase (sold, fluid or gas) of the canals in OUMNH PAL-J.028331 were to be changed, this might have an impact on the overall elastic constants (macroscopic properties) of the entire cortical bone. The solid would simulate the absence of canals, just homogenous bone. The liquid would simulate an environment where the canals are filled with tissue or blood, and the gas would simulate a situation where the canals were empty space.

The weight difference between solid, liquid and gas is substantial, with liquid filled canals offering 5% less mean density than the solid, and gas 16% less density than the solid. The macroscopic elastic properties of the bone could be predicted, assuming the behaviour would be homogenous through the cuboidal section of the cortical bone that was digitally extracted from the cortical bone. On average the Young’s Modulus decreased by 4.1% when liquid canals were used instead of solid bone (Fig. 5), although when gas canals were used instead, the Young’s Modulus decreased less, on average only by 3.1% (Fig. 5). Notably, the longitudinal Young’s Modulus, E_z_, is seemingly unaffected by the change from liquid to gas. The shear modulus was largely unaffected by the difference in liquid and gas, where the change from solid to liquid and solid to gas caused a 4.3% and a 4.2% reduction in shear modulus respectively. It is noteworthy that G_rθ_ decreased by more than 6% in both liquid and gas cases, which was the largest change caused by the canals (Fig. 5) (see Supplementary Table S1 Online). A less obvious pattern emerged from the Poisson’s ratio, which regardless of canal phase, increased on average by 2.1%, with gas affecting the rθ plane more and liquid affecting the θz plane to a greater extent.

## Discussion

This study aimed to show through both microscopic and macroscopic X-ray CT and subsequent numerical modelling that palaeobiological structures found in pterosaur bones could be spatially resolved at sufficiently high resolution to inspire, create, and/or refine efficient/lightweight components for industry. The gross morphology scans of OUMNH PAL-J.028331 on the Nikon XT H were not able to acquire the detailed porosity of the cortical bone, resulting in a microstructurally homogenous representation of bone that is typical of previously published CT scans. However, these macroscopic scans were helpful when postulating conclusions based on the Region of Interest (ROI) scans.

The non-destructive ROI scan images from the Zeiss Versa systems revealed higher spatial resolution that permitted direct comparison with more traditional (destructive) histological data^22,23^. The multi-scale imaging of the *Rhamphorhynchus* (OUMNH PAL-J.028331) phalanx revealed additional information that can only be gained from microscopic scans of large pterosaurs, but not easily discerned from destructive physical sectioning techniques (i.e. histological approaches). The outer layer of the pterosaur bone was largely intact but does show some evidence of damage and cracking (Fig. 1a), most likely due to taphonomy. The environmental conditions, specifically temperature and relative humidity, of the Museum’s Stores are stable enough to be an unlikely cause of deterioration of the specimen. The large crack in OUMNH PAL-J.028331 has split both the bone and the encasing sediment, meaning it was sustained post-fossilisation. It may have occurred when the bone was being extracted from the matrix in the 19th century, or during subsequent preparation into the small 132 mm x 41 mm x 14 mm sized block. The absence of any secondary mineralisation in the crack supports that it occurred after the fossil had been excavated. Unfortunately, this means no information can be gathered from this structure with regard to crack propagation in live pterosaurs. However, healed pathologies in pterosaur bones may in the future provide such data.

The relative density of the surface layer to the main cortical bone of OUMNH PAL-J.028331 cannot be determined from these scans as the effects of beam hardening (when an X-ray beam travels through an object, the low-energy photons are absorbed more than the high-energy photons) results in the edge appearing to have a higher attenuation coefficient (and thus a higher apparent density)^48^. The surface bone layer of OUMNH PAL-J.028331 has a thickness of ~ 60 μm on average (Fig. 2b). When compared with traditional histological thin-sections of other pterosaur species, the surface layer is comparable to the periosteal layer of those specimens, suggesting preservation of the periosteum in OUMNH PAL-J.028331^49–51^. There were no obvious Lines of Arrested Growth (LAGs) observed in OUMNH PAL-J.028331, but these have been previously recorded in pterosaur histology^49,50^. The absence of LAGs was most likely due to a higher contrast required to resolve such features that may be of similar density to the adjacent bone matrix. LAGs might be possible to image if lower energy X-ray photons were employed. However, the current study needed higher energy photons to penetrate the encasing sedimentary matrix to image the bone.

The main achievement of this study was the acquisition of adequate image quality to spatially resolve fine-scale (pixel size of 2 μm) bone microarchitecture. There was a lack of obvious trabecular structure in OUMNH PAL-J.028331 scans, though this is unlikely to be a function of the noise or pixel size, as the Zeiss scans show structures that are smaller than known trabeculae dimensions^30,52^. Therefore, it is possible that trabecular structures were not present in this region of bone, they were destroyed during the fossilisation process, or the trabeculae had a similar enough attenuation to the sediment that they were indistinguishable using X-rays due to similarities in contrast. Two of the main factors affecting image quality are flux and pixel size. A higher flux means more X-rays are penetrating the sample and reaching the detector, increasing the signal relative to the baseline noise, thereby creating a clearer image^53^. At a constant energy, current and exposure time, the only way to increase flux is by moving the detector closer to the source. However, this increases the pixel size and subsequently reduces the level detail that could be resolved. Some ROI scans of the proximal head of OUMNH PAL-J.028331 were attempted but its encasement in the sedimentary matrix meant that insufficient flux could be achieved for pixel sizes that would result in an adequate resolution to resolve a trabecular structure. Ideally, a fossil specimen should be isolated (removed entirely) from any embedding sedimentary matrix when attempting the visualisation of trabecular structure, but the historical and scientific value of the material being scanned meant this level of preparation was not possible for OUMNH PAL-J.028331.

The most significant structures identified during the scanning of OUMNH PAL-J.028331 were the multiple canals resolved in the cortical bone. The canals were mapped in 3D, allowing their distribution to be non-destructively mapped three-dimensionally and quantified on a larger scale, unlike the more traditional 2D mapping of canals through the destructive thin-section-based histology. The canal porosity of OUMNH PAL-J.028331 constitutes 16% of the total bone volume.

This value of 16% for the cortical bone porosity can be observed in very young birds^54^ but is higher than that observed in adult birds^54^, humans^55^, and other fossilised archosaurs^56^. Why this sample is an outlier is unknown, as some of the porosity might be a consequence of the alteration/decay of non-mineral components of the bone during fossilisation. Further scans of pterosaur material could help determine whether this porosity is a function of species or the age/health of the pterosaur. Notably, the average vascular diameter of 10.6 μm is in agreement with the vascular diameter of other pterosaur species^57^, which is more similar in size to small mammals than small birds^54,58^.

While there is not sufficient contrast to show individual primary or secondary osteons, the pores are likely to be predominantly vascular canals based on their morphology, location, distribution, and geometry^49–51,59^. Assuming these are vascular canals, they would be filled with fluid (blood) and other tissues (nerves) necessary for bone function^60^. The presence of the pores are undoubtedly a biological necessity, but their potential to influence the mechanical properties and behaviour of bone can be evaluated using Finite Element (FE) modelling. Martin and Palmer^61^ showed that X-ray CT can be an excellent tool for quantifying pneumaticity and volumetric approximations in pterosaurs, so when accompanied by additional information provided by multi-scale imaging, these estimates can be further refined.

The measurable shift in this preliminary model suggests that the bone becomes 4.1% less stiff when liquid canals are present, versus the 3.1% decrease when gas canals are present. Gases are more easily compressed than liquids because of their lower density, so when a force is applied to bone and the canals are compressed or stretched, it follows that the bulk cancellous bone with less dense gas canals would be stiffer than bone filled with liquid. However, it is extremely unlikely that these canals in pterosaurs were uniquely filled with gas instead of fluid and other tissues as it would produce some biological challenges. However, there is the possibility to reverse engineer these canals for aerospace applications, by introducing gas-filled canals of similar dimensions to populate manufactured materials that might offer benefits in terms of weight reduction. This would be a ~ 3% reduction in stiffness coupled with a ~ 16% reduction in density to the structure. The geometry and microarchitecture of palaeontological materials might also provide a reasonable starting point for the optimisation of manufactured components, given the evolution of the biomaterials has undergone an evolutionary iterative process that is comparable to engineering design.

Therefore, we propose that this fine-scale porosity provides MicroArchitecture Accommodation Capacity (MAAC) to a material. We define this new term as a microscopic geometry permitting additional functionality without compromising the macroscopic properties of the original structure. For example, if MAAC is used in aerospace components, the fundamental material properties of the metal alloys and the composite materials used in construction would remain largely unchanged, but self-healing foams or sensors could be inserted into the space created by the MAAC. The network-like architecture of the pterosaur canals provides accommodation space when the bone deforms and is a clear example of when the MAAC impacts the behaviour of a material.

An increase in porosity also appears to lower bone stiffness, though this is a simple preliminary model that does not account for the potential impact of collagen and other proteins present in bone. The FE model assumes homogenous bone (aside from the canal microarchitecture) with only a mineral phase, whereas in real bone the organic phase (collagen and other proteins) increases stiffness^62,63^. A model that combines both the mineral and organic components of bone would more accurately represent this biomaterial in stressed environments, but the results in this study clearly suggest that the borrowed microarchitecture resolved from fossil bone can still impact the design of engineering components. In reality, the porosity found in this study is but one part of a greater whole that makes bone an effective, yet illusive material. Future developments in imaging techniques and further measurements of pterosaur material should yield a better picture of how bone porosity interacts with trabeculae and other possible factors at the micro-structural level. Understanding the effect of microstructure on pterosaur flight will contribute to even more efficient pterosaur-inspired engineering components.

Combining the imaging data and FEA results from this study, it is possible to suggest there is an optimum porosity for manufactured bioinspired composites that would improve function and mechanical strength. These optimums will potentially be material-specific, so results from fossil bones might well provide a starting point for the reverse engineering evolution of new materials. Therefore, a microarchitectural functional bracket might additionally help optimise materials that impact the locomotion behaviour of animals^64^ but also the manufactured components derived from such structures. This work might also impact how we view diseases that reduce the structural stability of bone (e.g. osteoporosis) given that drastically increased porosity adversely affects structural integrity^65^. Industries that demand high-performance and low-weight components (e.g. automotive and aerospace) could benefit from a palaeo-biomaterial-based approach to better understand the reduction in elasticity (with increased flexibility), crack deflection^42^ and stress mitigation from the simulations of fossilised bone conducted in this investigation. Future bioinspired components could integrate canal-like structures inspired by vascular canal networks that could be optimised through modelling to meet the specific requirements of engineered components. Therefore, if the structure and microarchitecture of bone (both extant and extinct) are quantitatively resolved and tested using numerical models, it might just provide the next step in devising solutions to the engineering challenges that face humanity in the 21st century.

## Methods

### Sample preparation

The pterosaur *Rhamphorhynchus indeterminate* OUMNH PAL-J.028331 phalanx I of digit IV (‘wing’), was loaned by Oxford University Museum of Natural History (OUMNH) in the UK (see Supplementary Fig. S1). The first phalanx was chosen for the focus of this study because it is the midpoint for much of the load in the pterosaur wing. Unlike birds and bats, which translate load through a variety of digits (some fused in the case of birds), pterosaurs translate a large proportion of the mid-distal wing load through a single digit (digit IV)^66^. The first phalanx is an integral, if not pivotal focus of load during flight. The specimen was excavated from the Stonesfield Slate (Taynton Limestone Formation) from the middle Bathonian (~ 167 million years old)^67^. Rhamphorhynchus sp., was not the largest of pterosaurs and its tailed body was relatively conservative but the skulls of this genus were diverse in form, suggesting high plasticity and possible niche partitioning. As pterosaurs evolved into much larger forms in the late Jurassic and into the Cretaceous, the loss of a tail and increase in body, wing and skull size was dramatic, which again points towards a diverse group of volant animals well-adapted to their environments^68^. Following standard museum practice, permission was obtained from the Collections Manager (co-author on this paper) at the Oxford University Museum of Natural History to gain access to the fossil material used in the study. All specimens loaned and studied in the course of this research have been returned to the Oxford University Museum of Natural History.

The sample was mounted for CT analysis in such a way as to prevent movement. For the Nikon XT H scan, a cup was made out of florist’s foam, a low attenuating material, filled with silica gel beads. The beads help reduce the beam hardening effect that happens when low-energy photons are preferentially absorbed by the surfaces of materials^48^. For the Zeiss Scans, a 3D printed cylinder was used with foam placed around the bone. In both cases, the bone was wrapped in clingfilm to protect the sample and to help in the segmentation process by separating the beads from the bone.

### X-ray CT setup and parameters

The source was adjusted to ensure a new tungsten surface was being impacted by the electrons, therefore reducing the effects of X-ray scatter and anisotropic X-ray production from micro-craters in the source target. This can be done manually for the Nikon XT H and via the software on Zeiss machines. The magnets inside the electron tube were adjusted to ensure a sharp focal spot and minimise both the anisotropy of the spot shape and focal spot blurring.

After auto-conditioning left the X-rays stable enough for scanning, the specimens’ locations were optimised by monitoring the flux of X-rays through the specimens at different positions within the CT system, aiming for a balance between pixel size and flux. This was a complex process with the pterosaur fossil as the sedimentary matrix created variations in attenuation thickness (see Supplementary Fig. S2). The rectangular nature of the sediment block left intact by the preparator created very high transmission through the thin areas and very low transmission through the thick areas. To overcome this issue, Lowe developed a method using glass beads to surround the sediment. The beads absorbed more X-rays in thinner regions, in addition to absorbing low-energy X-rays that would cause beam hardening artefacts. Once the X-rays have passed through the target, the beads on the other side reduce scatter by re-absorbing the low-energy X-rays. Naturally, the beads and sediment meant that a high energy, current and exposure time were needed, leading to a much longer scan (see Supplementary Table S2). For the Zeiss scans of the pterosaur, a protruding part of the cortical bone was exposed slightly beyond the sediment. This allowed for a 1.986 μm pixel-size scan of the cortical bone. Once the parameters had been established, one final step before scanning was required: using a reference object, also known as an Image Quality Indicator (IQI). The usage of an IQI (or phantom) is not common practice in palaeontology, though phantoms are frequently used in medicinal research. This is done by creating features of set sizes that are verified with microscopy, so the sizes of the features in the CT virtual volume can be compared to their verified sizes. Therefore, IQIs provide a ground truth that ensures that the features seen are real and not artefacts of the data collection process.

**Fig. 6:**
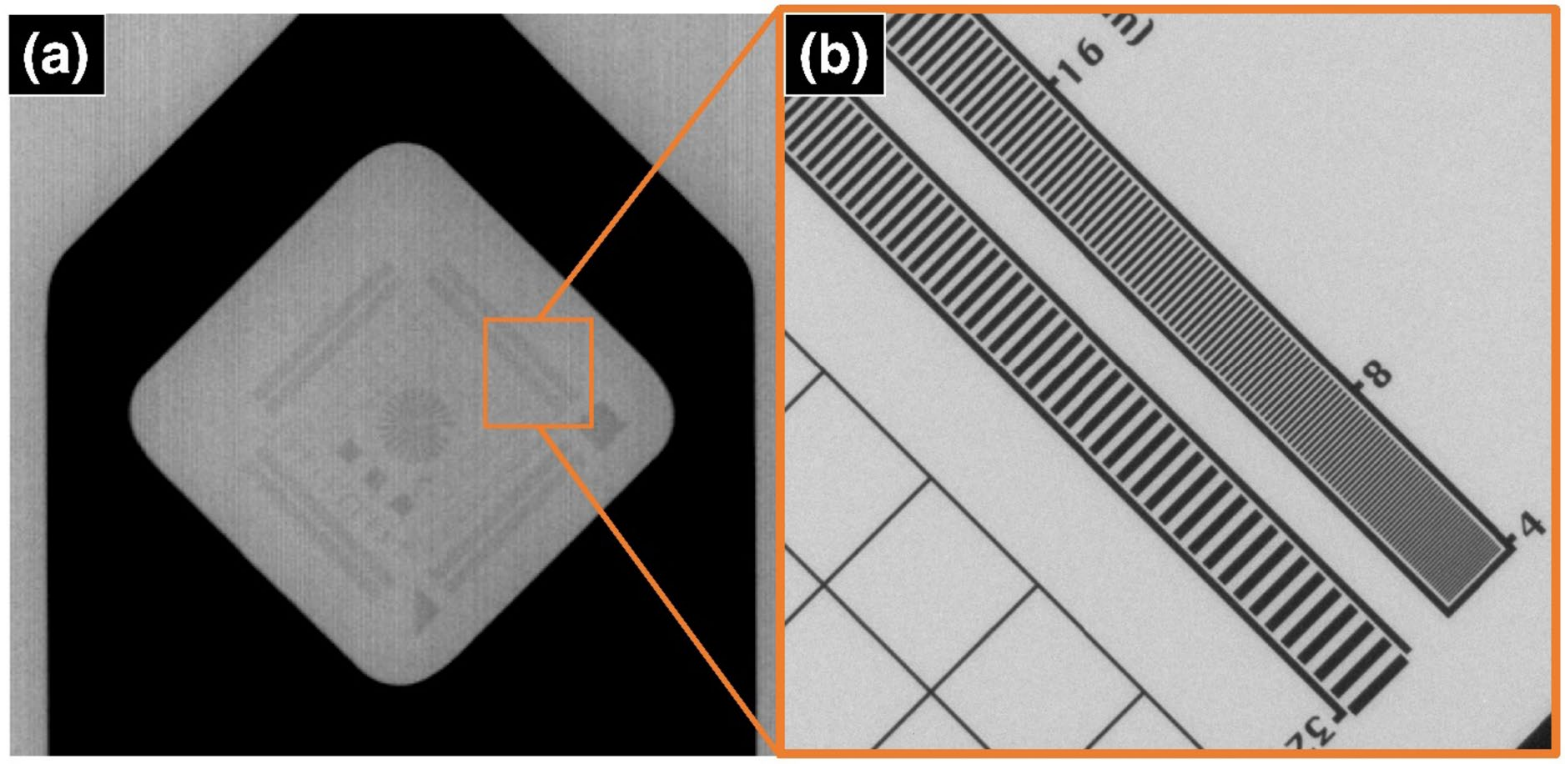
The Zeiss Star Image Quality Indicator (IQI) that was used as a reference for different resolutions at different pixel sizes. The Zeiss Star is made of 8 different gratings that each span from 32 to 4 μm, (**a**) shows the IQI on a Nikon XT H with a pixel size of 50 μm, and (**b**) shows the square in (**a**) on a Zeiss 520 Versa with a pixel size of 2 μm. The square in (**a**) shows the region that is seen in (**b**), demonstrating the significant drop in resolution.

The IQI chosen for this experiment was the Zeiss Star IQI (Fig. 6), manufactured by Zeiss and used during the installation process. It was mounted into both the Nikon and Zeiss CT machines after the settings for each scan were established. Radiographs were taken at each setting to find the threshold at which the lines in the grating begin to look like one blurred object and stop appearing visibly distinct. The last distinct line is defined as the minimum resolvable distance (or resolution), which was quantified by the Aletheia Imaging Solutions software package^69^. Aletheia allows the user to input an image of any IQI and once aligned, outputs the resolution of the image using the Modulation Transfer Function (MTF) limit using the guidelines in ASTM standard E2002-15^53,70^. This data informed the later segmentation processes and the modelling of the bones for FEA.

### Post-processing

The data was reconstructed from the respective systems without the use of corrections (beam hardening, scatter, drift etc.). Due to the use of glass beads, beam hardening wasn’t an issue, and other corrections would change the legitimacy of the data. The orthoslices were imported into Thermofisher’s Avizo software^71^. This allowed for the reconstructed slices to be segmented, though the low contrast between the sediment and bone prevented a pure automatic segmentation method. Non-localised noise filtering was used to remove some of the noise, and then automatic Hysteresis thresholding was used to find a rough outline, although some small speckles appeared in the bone itself. Manual segmentation was therefore employed to ensure noise hadn’t affected the recognition of some key features (see Supplementary Figs. S3, S4). This involved grouping pixels of similar grayscale value and manually selecting what features were bone and what were canals. The *Rhamphorhynchus* (OUMNH PAL-J.028331) phalanx fossil was segmented into bone, sedimentary matrix and canalicular features. Any canals or pores that had a diameter smaller than the spatial resolution determined using the Aletheia Software and the IQIs were removed.

**Fig. 7:**
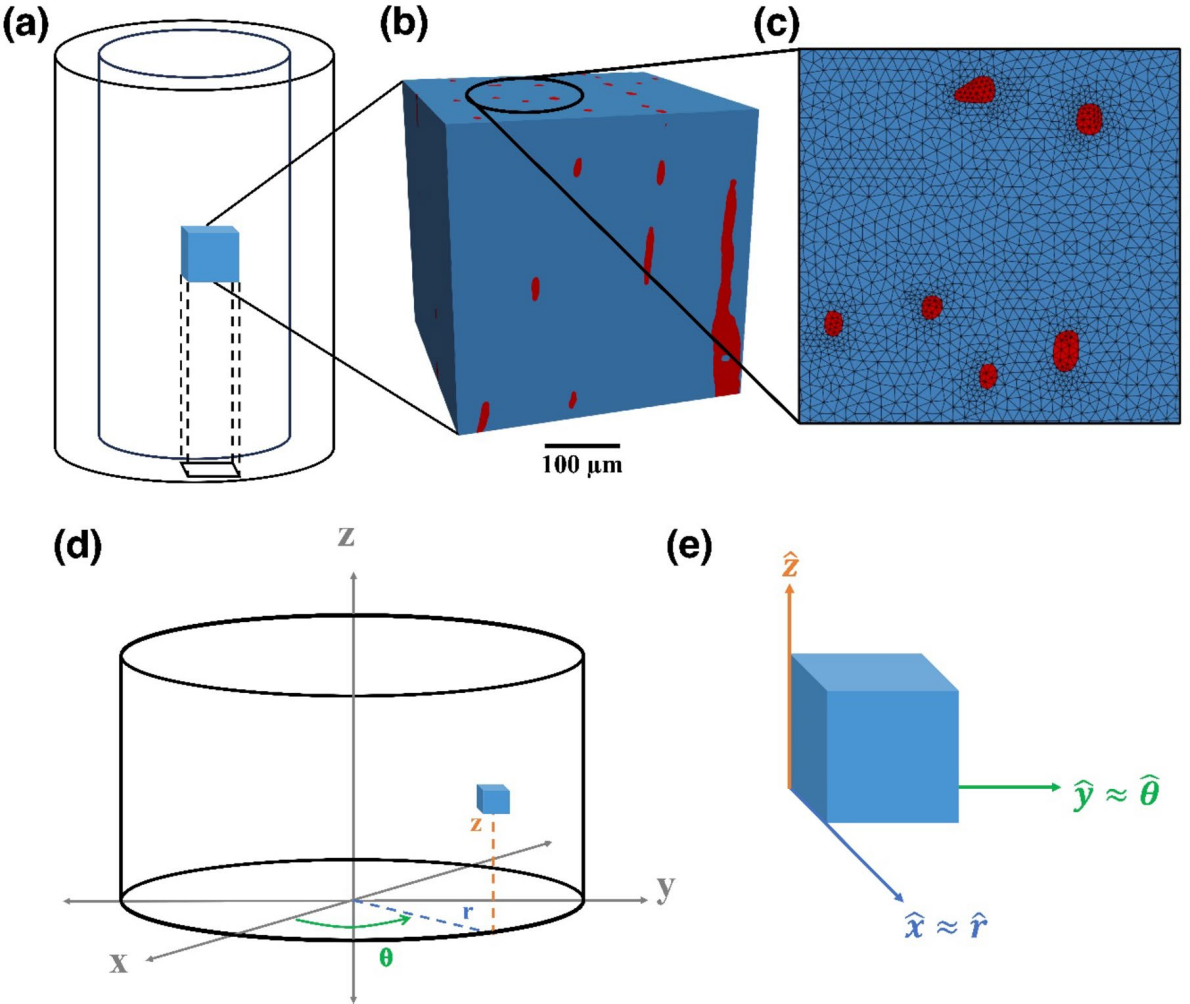
The Finite Element model that was used to simulate the effects of stresses on the cortical bone of the *Rhamphorhynchus* (OUMNH PAL-J.028331) phalanx, generated using Simpleware ScanIP S-2021.06^72^. The model is cuboidal in shape, where the blue areas are modelled as bone and the red areas had gas, solid and liquid phases modelled. The results assume that the cortical bone forms a cylinder, (**a**) showing the cube’s placement and orientation in the bone. (**b**) Shows the model’s size and distribution of canals, and (**c**) shows the meshing of the individual elements on the top face on the z-axis. (**d**) Shows the element’s place in a cylinder (representing the bone), and (**e**) shows how the terminology for the final variables was established, based on a small element in a cylinder. The symbols xyz are cartesian coordinates and rθz a re cylindrical coordinates. Symbols with the hat (circumflex $\: \widehat{x}$) are a mathematical notation for the unit vectors (‘in the direction of’). Thus $\:\widehat{x}\approx\:\widehat{r}$ means that for this small element the x direction is equivalent to the r direction.

These canals span the entire cortical structure and were modelled as three different phases of matter: gas, liquid and solid (Fig. 7). This was to investigate the effects of changing material properties of micro-structures on the macroscopic properties of the *Rhamphorhynchus* (OUMNH PAL-J.028331) phalanx. The mesh found in Fig. 7 was created using Simpleware ScanIP^72^, making sure that no element was smaller than the resolution determined by the IQI. The software used for the FEA was the Multiphysics Object-Oriented Simulation Environment (MOOSE) developed by Idaho National Labs^47^. MOOSE has seen use in nuclear reactor safety studies^73,74^. It can solve FE problems in parallel, scalable up to 30,000 cores. For the multi-million element meshes produced by this study, such powerful software was required.

The approximate values for the elastic properties of the pterosaur bone were estimated from a review of cortical bone from various species (including alligator jaws, mammalian long bones and bird wing bones)^75–80^ (see Supplementary Table S3). A review of these sources showed that many cortical structures had similar elastic properties in their longitudinal directions (parallel to the central canals). We chose our values as an approximation, with a focus on the difference between the non-canal model and the canal-based model, and less of a focus on the raw values.

The values for the canals are reasonable order of magnitude estimates, chosen relative to the values used in the solid phase and based on engineering judgement. They are not from a published source. In a mixed-phase model of this kind, a precise set of values for liquid and gas is not required to demonstrate the relative responses of the canals filled with solid, liquid and gas. The value for the gas would be zero. However, if zero was applied, the FEA software would fail to find a solution. In such a case, the FEA expert would apply a small number which could be said to be effectively zero. This approach is accepted practice as FEA is a modelling technique that gives engineering insight rather than definitive answers. For a prototype palaeo-inspired engineering material, experimental work would be carried out to validate the model and the choice of parameter values.

While cortical bone is known to be elastically anisotropic, this was a basic model that was looking more into a comparison between three states, the magnitude of the elastic moduli was less important than the effect of the canals on the stresses. The model of the bone was constrained on the bottom face along the z-axis, and a strain was applied on the top face. The stresses measured were the von Mises to aid in visualisation, and the principal stresses were measured in the direction of strain. For a qualitative investigation into the effects of the canal phase on the stresses on the cortical bone, a strain of 0.02 (i.e. 2% of the length of the bone sample) was applied in three scenarios; where the canals were treated as solid, liquid, and gas. This gave some initial insight into the behaviour of bone in the longitudinal axis, although to complete the full description of this material the same process was repeated along different axes to obtain the necessary elastic constants^81^ (see Supplementary Table S4). The elastic properties are denoted as longitudinal, radial, and azimuthal, as the properties of the entire cortical bone can be estimated as the same as this small element’s properties—assuming a cylinder with homogenous canal patterns angularly (Fig. 7d, e). Hence the x-axis aligns along the radius for a small angle, y follows the azimuth for a small angle, and the z-axis remains the same. With these experiments, Young’s modulus, Poisson’s ratio, and shear modulus were found for each of the planes in the three scenarios where the canals were treated as a solid, liquid and gas.

## Electronic supplementary material

Below is the link to the electronic supplementary material.

Supplementary Material 1

## Data Availability

As the X-ray CT datasets presented are large, the data will be stored by the University of Manchester’s Research Data Management (RDM) service, which satisfies the UK Research Council’s RDM guidelines. Furthermore, full accessibility will be granted upon request (via email) to the corresponding author.
