# Supplementary material for: Harnessing 3D microarchitecture of pterosaur bone using multi-scale X-ray CT for aerospace material design
Source: Sci Rep. 2025 Feb 17;15:5719. doi: 10.1038/s41598-025-88257-0 (PMC11833079; doi:10.1038/s41598-025-88257-0)
Supplement: Supplementary file 1 — Supplementary Material 1 [file 41598_2025_88257_MOESM1_ESM.pdf]

Harnessing 3D microarchitecture of pterosaur bone using multi-scale X-ray CT for aerospace material design.

Nathan Pili[1,*], Tristan J. Lowe[2], Lee Margetts[3], Kevin Pickup[4], William I. Sellers[1], Emma L. Nicholls[5], Philip J. Withers[2], and Phillip L. Manning[1]

[1] The University of Manchester, Department of Earth and Environmental Sciences, Manchester, M13 9PL, UK
[2] Henry Royce Institute, The University of Manchester, Department of Materials, Manchester, M13 9PL, UK
[3] The University of Manchester, School of Engineering, Manchester, M13 9PL, UK
[4] BAE Systems, Samlesbury Aerodrome, Balderstone, BB2 7LF, UK
[5] Oxford University Museum of Natural History, Parks Road, Oxford, OX1 3PW, UK

[*]nathan.pili@postgrad.manchester.ac.uk

Supplementary Information

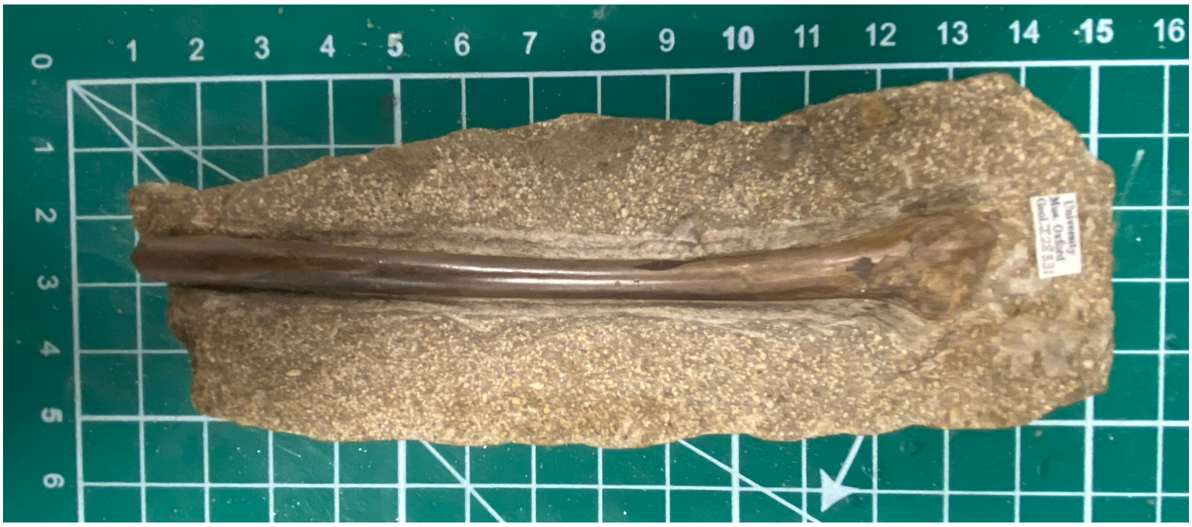

Figure S1 – Sample OUMNH PAL-J.028331. The *Rhamphorhynchus* phalanx that was scanned. The scales are in cm.

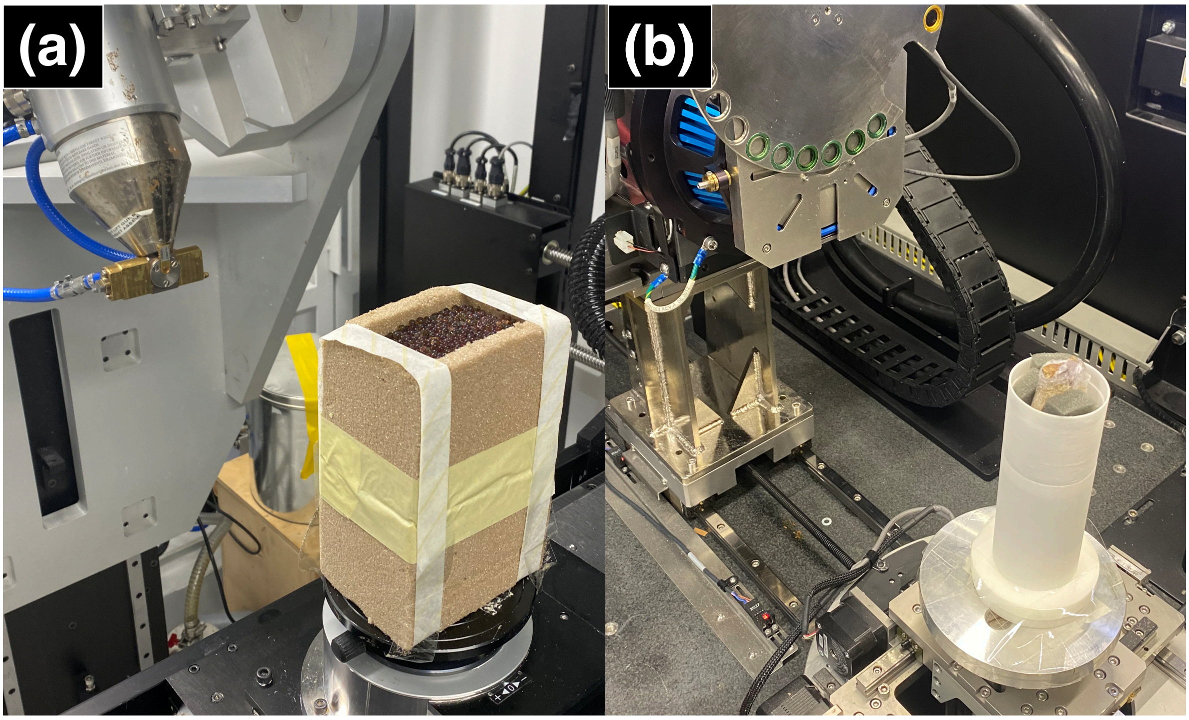

Figure S2 – CT Geometry and Setup. The setup for the Nikon XT H (a) and Zeiss Versa (b) instruments. The container was changed between scans to allow the source to get closer to the Zeiss instrument, thus increasing resolution by reducing pixel size.

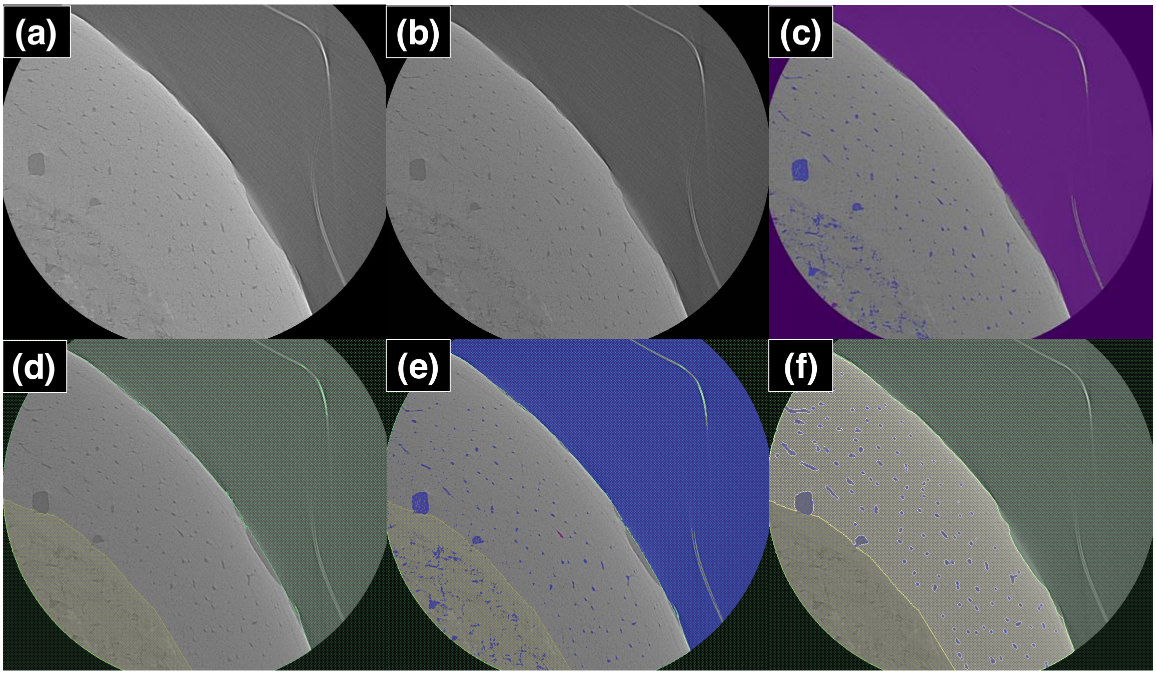

Figure S3 – Segmentation Process. The different stages of manual segmentation undertaken using Avizo software. (a) Shows the raw image, and (b) the same orthoslice after noise filtering. (c) the selection of the background and air in purple. (d) the air and black background (now in green), and the sediment in yellow at the centre of the bone. The sediment was cut out from the bone through the tracing tool in 3D. (e) Shows a pore selected in purple, which is repeated for every pore in 3D. (f) Shows the final product of segmentation, with the background in green, the sediment in yellow, the bone in white, and the pores in blue.

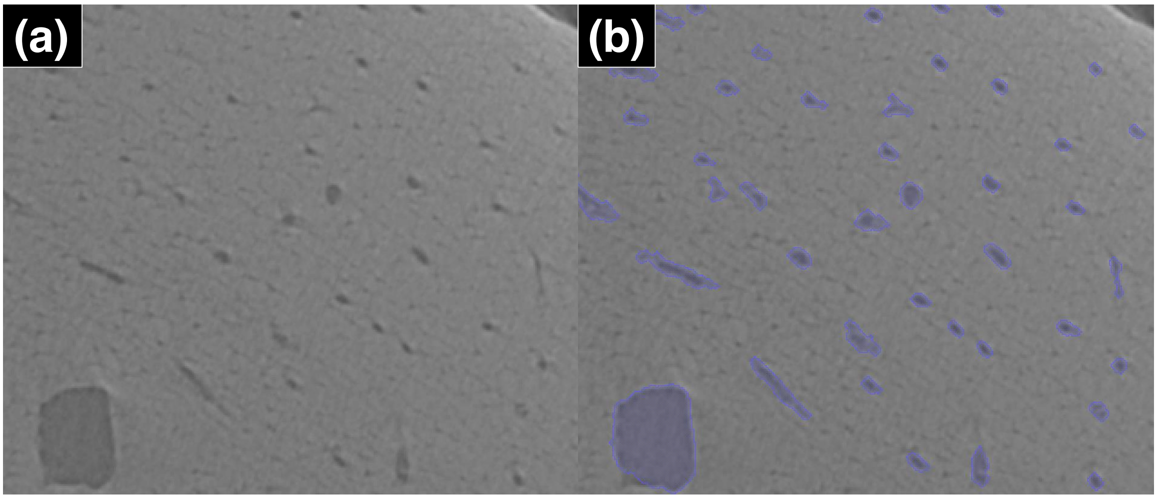

Figure S4 – Segmentation Review. How the process reflects the data. (a) shows the raw data, and (b) shows the overlay of the segmented sections. The method has resulted in some segmented areas that are a few pixels larger than the porosity, but this was to compensate for the low contrast. Any pore that was below the established resolution was removed.

Table S1 - The FE Results. The results of the various displacement tests. The final variables are given in cylindrical coordinates representing the homogenised properties of the cortical bone modelled as a cylinder. E_i is Young's modulus in the i direction, ν_{ij} is Poisson's ratio in the ij plane, and G_{ij} is the Shear Modulus in the ij plane.

Canal Phase	Solid	Liquid	Gas
E_r (GPa)	20.000	19.117	19.320
E_θ (GPa)	20.000	19.069	19.465
E_z (GPa)	20.000	19.387	19.388
$\nu_{r\theta}$	0.37000	0.37466	0.38177
ν_{rz}	0.37000	0.37725	0.37566
$\nu_{\theta z}$	0.37000	0.38125	0.37646
$G_{r\theta}$ (GPa)	14.599	13.659	13.720
G_{rz} (GPa)	14.599	14.096	14.091
$G_{\theta z}$ (GPa)	14.599	14.155	14.156

Table S2 – CT Parameters. The instrument parameters for the *Rhamphorhynchus* (OUMNH J28331) phalanx scan on the Nikon XT H and Zeiss Versa Systems.

Machine:	Nikon XT H 225	Zeiss Xradia 520 Versa	Zeiss Xradia 620 Versa
Subject:	J28331 - Entire Specimen	J28331 - 2mm of tip	J28331 - 2mm of tip
Energy:	225 kV	140 kV	160 kV
Power:	50 W	10 W	25 W
Exposure time:	1.42 seconds	10 seconds	Scan 1: 5 seconds<br>Scan 2: 60 seconds
Frame averaging:	4 frames	1 frame	1 frame
Binning:	2x2	2x2	2x2
Projections:	2501	3201	3201
Effective Pixel Size:	50 µm	1.986 µm	Scan 1: 1.925 µm<br>Scan 2: 0.799 µm
Filter:	2mm of copper	None	Zeiss LE1
Optical Magnification:	N/A	4X optics	Scan 1: 4X optics<br>Scan 2: 10X optics

Table S3 – FE Parameters. The variables used in the FE simulations, specifically what was changed to the canal properties to simulate the phase change.

Canal Designation	Solid	Liquid	Gas
Bone Poisson's Ratio	0.37	0.37	0.37
Bone Modulus (GPa)	20	20	20
Bone Density (kgm^{-3})	1500	1500	1500
Canal Poisson's Ratio	0.37	0.49	0.4999
Canal Modulus (GPa)	20	0.2	0.02
Canal Density (kgm^{-3})	1500	1000	1

Table S4 - FE Geometry. The different displacement cases and what information was obtained in these scenarios.

Case Number	Model	Boundary Conditions	Elastic Constants Found
1	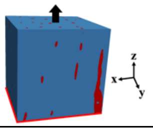	Displacement of top xy face in z. Constraint of the bottom xy face in xyz.	E_z<br>ν_{rz}<br>$\nu_{\theta z}$
2	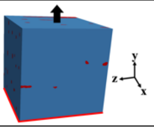	Displacement of top xz face in y. Constraint of the bottom xz face in xyz.	E_θ<br>$\nu_{r\theta}$
3	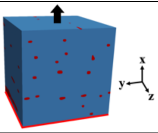	Displacement of top yz face in x. Constraint of the bottom yz face in xyz.	E_r
4	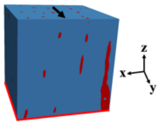	Displacement of top xy face in y. Constraint of the bottom xy face in xyz.	$G_{\theta z}$
5	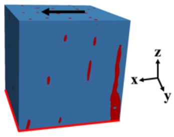	Displacement of top xy face in x. Constraint of the bottom xy face in xyz.	G_{rz}
6	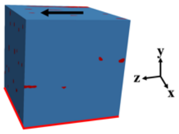	Displacement of top xz face in x. Constraint of the bottom xz face in xyz.	$G_{r\theta}$
